# The impact of mindfulness therapy combined with mentalization-based family therapy on suicidal ideation in adolescents with depressive disorder: randomized intervention study

**DOI:** 10.1186/s12991-024-00503-3

**Published:** 2024-05-08

**Authors:** Xiao-Fen Fan, Ju-Yi Peng, Li Zhang, Ya-Li Hu, Yan Li, Yue Shi, Tian-Mei Zhang

**Affiliations:** Department of Mental Health, Shanxi Bethune Hospital, Shanxi Academy of Medical Sciences, Third Hospital of Shanxi Medical University, Tongji Shanxi Hospital, Taiyuan, 030032 China

**Keywords:** Mindfulness therapy, Mentalization-based family therapy (MBFT), Adolescents, Depressive disorder, Suicidal ideation

## Abstract

**Background:**

Adolescents with depression who engage in non-suicidal self harming behaviors are more likely to adopt negative coping strategies when faced with negative events. Therefore, these patients should be introduced to positive coping strategies. Evidences have showed that mindfulness-based interventions can positively impact the psychology of patients with mental disorders. This study was to explore the impact of a combination of mindfulness therapy and mentalization-based family therapy (MBFT) on suicidal ideation in adolescents with depressive disorder.

**Methods:**

Eighty adolescent patients with depression and suicidal ideation admitted to our hospital from September 2021 to February 2022 were selected as subjects. They were divided into a control group and a study group using the random number table method, with each group comprising 40 subjects. The control group received MBFT, whereas the study group received both mindfulness therapy and MBFT. The psychological status and suicidal ideations of the two groups were compared before and after the intervention.

**Results:**

The psychological health scores of both groups of patients were lower after the intervention, with the scores of the study group being lower than those of the control group (*P* < 0.05). The scores on the suicidal ideation scales for both groups were lower after intervention, and the study group scored lower than the control group (*P* < 0.05). The absolute values of the differences in psychological health scale scores and suicidal ideation scale scores before and after the intervention were higher in the study group than in the control group (*P* < 0.05).

**Conclusion:**

The combination of mindfulness therapy and MBFT can improve the psychological condition of adolescents with depression, reduce their suicidal ideations, and help them develop a healthy and positive outlook toward life, making this method worthy of clinical recommendation.

## Background

Mental health of adolescents is important for their as well as their families’ well-beings, and it also closely influences the future of the society. Teenage suicide is a global mental health issue. The World Health Organization has found that suicide is the third leading cause of death among adolescents aged 15 to 18. Most adolescents with suicidal ideation and behavior seem to suffer from severe depressive disorder (MDD) [1, 2]. In China, the incidence and suicide rates of depression among adolescents are increasing every year. A survey showed that the prevalence of suicidal ideation among Chinese adolescents with depression is 38.2% (625/1635) [3]. Epidemiological studies have shown that nearly 20% of teenagers in the United States consider suicide each year, 15% of teenagers develop suicide plans, and nearly 10% of teenagers attempt suicide [4]. The characteristics of adolescence are rapid changes in the brain, pruning of excitatory synapses, and increased myelin formation in the entire frontal, temporal, and parietal regions to promote emotional regulation, impulse control, and executive function [5]. The US Food and Drug Administration (2003) defined this neurodevelopmental window as 12–21 years old, which is a period of great risk and opportunity [6]. In addition, the occurrence of suicidal behavior is one of the most common reasons why depressed adolescents start taking antidepressants. If left untreated, pre adolescent depression may persist into adulthood and even gradually worsen [7]. However, the efficacy of antidepressants is detrimental to suicidal ideation, as some MDD patients commit suicide before the medication takes effect.

Adolescents with depression who engage in non-suicidal self harming behaviors are more likely to adopt negative coping strategies when faced with negative events. Therefore, these patients should be introduced to positive coping strategies [8].

Inappropriate parental behavior has also been identified as a risk factor for suicidal ideation in adolescents with depression. The risk factors for adolescent suicide include depression and reduced family support [9]. The externalization behavior of depression often includes aggression, self harm, or suicide. Suicide ideation in adolescents is not only a symptom of depression, but also serves as a warning to the external environment through externalization behavior. Previous studies have shown that increased family conflict and decreased intimacy are factors influencing adolescent suicidal ideation [9]. Depression, anxiety, decreased self-esteem, insufficient family cohesion, and social support are predictive factors for the risk of attempted suicide among adolescents. To mitigate this, parents should provide more attention, understanding, and companionship to their adolescent children [7]. The increase in parental control leads to a significant increase in suicidal ideation among adolescents, while the more care and support from parents, the less suicidal ideation among adolescents. Parental care in parenting relationships can reduce the risk of depression and suicidal ideation in adolescents, while parental control can increase the risk of depression and suicidal ideation in adolescents. Therefore, in family upbringing relationships, building a caring parenting relationship with parents, reducing excessive control behavior, and enabling children's emotions to be effectively self managed can reduce the occurrence of depression and suicidal ideation in adolescents. Parents should pay attention to maintaining their emotional stability, provide more encouragement and care to teenagers, avoid excessive control, and reduce the occurrence of depression and suicidal ideation in teenagers [7].

Psychologically based psychotherapy is a psychodynamic therapy rooted in attachment and personality theory [10]. It aims to enhance patients' ability to understand their own and others' psychological states in an attachment environment, in order to address their difficulties in emotional, impulse regulation, and interpersonal functions [11]. Psychotherapy was originally aimed at treating borderline personality disorders [27], but now it is used to treat various types of mental disorders such as depression, eating disorders, drug abuse, and other types of personality disorders [11]. Mentalization-based therapy (MBT) was initially developed for adults with borderline personality disorder [12], and subsequent studies by Griffiths et al. [13] and Bo et al. [14] have demonstrated its positive effects on suicidal ideation in adolescents with depression. Mindfulness, widely practiced in Buddhism, can be conceptualized as a specific form of focus [15, 16]. MBT was originally developed to treat adults diagnosed with borderline personality disorder (BPD), and it directly addresses the fragile psychological abilities that are core features of BPD and other complex pathologies [10]. In the adult BPD population, MBT reduces self harm, emotional distress, and hospital stay compared to normal treatment, and improves social function, with continuous improvement at 18 months [17, 18]. In adult outpatient settings, MBT is also associated with reducing self harm [11]. For the adolescent population, MBT is more effective in treating self disabled adolescents than traditional treatment [19]. The treatment plan includes weekly individual MBT-A courses and 12 or more MBFT months. In a uncontrolled trial, self-reported self harm was reduced in the context of a reinforced MBT group program targeting adolescents with BPD or subthreshold BPD, which included introductory psychological education group meetings, 34 MBT group treatments, and 7 MBT parent (MBT-P) meetings [14]. Therefore, it is rational to believe that family therapy courses focus more on the process and aim to promote psychologization among group participants.

Mindfulness refers to a process that leads to a mental state, characterized by non critical awareness of a person's current experiences, including their feelings, thoughts, physical states, consciousness, and environment, while encouraging openness, curiosity, and acceptance. The two components of mindfulness have been distinguished, one involving self-regulation of attention, and the other involving orientation towards the present characterized by curiosity, openness, and acceptability. The basic premise of mindfulness practice is that experiencing the present in a non critical and open manner can effectively combat the influence of stressors, as excessive tendencies towards the past or future when dealing with stressors may be related to depression and anxiety. Several meta-analyses [20–23] have found that mindfulness-based interventions (MBI) can positively impact the psychology of patients with mental disorders. Mindfulness intervention may cause anxiety, discomfort, or even dissociative symptoms in patients [24]. Previous reviews generally suggest that mindfulness-based interventions (MBI) may help alleviate stress, anxiety, and depression. However, the vast majority of these reviews are qualitative and do not quantify the magnitude of therapeutic effects [25–27]. In contrast, only a few reviews have used meta-analysis methods to quantify the efficacy of this treatment. One review focused on MBI reducing stress in cancer patients [28], while another study investigated the effects of mindfulness therapy on pain associated with general physical or mental issues such as chronic pain, coronary artery disease, and fibromyalgia [29]. The results of these reviews are encouraging, indicating that MBI is moderately effective in reducing pain associated with physical or psychosomatic diseases. However, both reviews are based on a small number of studies, with relatively small sample sizes for each study. Two reviews dedicated to studying the impact of MBI on emotional and anxiety symptoms have yielded different conclusions.

Based on this, we applied both psychotherapies (MBI and MBFT) to adolescent patients with depression who had suicidal ideation; we aimed to investigate the impact of this combined therapy on the patients’ suicidal ideation and psychological well-being.

## Subjects and methods

### Subjects

A total of 80 adolescents with depression and suicidal ideation admitted to our hospital from September 2021 to February 2022 were selected as study subjects. The inclusion criteria were as follows: (1) fulfilling the diagnostic criteria for adolescent depression in the International Classification of Diseases, 10th edition (ICD-10) [30]; (2) aged between 12 and 18 years; (3) having elementary school level or higher education, fluency in reading and writing Chinese, and being able to independently answer the survey questions; and (4) being in the recovery phase of the disease (determined using Montgomery-Åsberg Depression Rating Scale (MADRS) score and working memory improvements) [31]. Necessary therapy was employed for the particpants during the study (since the participants were in recovery phase, pharmacotherapy was not applied and only the studing psychological therapy was utilized). Patients on the schizophrenia spectrum or having other psychotic disorders; addicted to alcohol or other psychoactive substances; having severe physical or brain illnesses; who were impulsive and uncooperative; and having intellectual impairments, making them unable to comprehend the content of the surveys, were excluded. Using the random number table method, the subjects were divided into a control group and a study group, with 40 patients in each group (simple randomization was applied). The baseline characteristics did not differ significantly between the two groups (*P* > 0.05) (Table 1). All patients were diagnosed with depression and severity of depression were matched between two groups. All patients involved in this study and their primary guardians were informed about the study, and both parties voluntarily signed the Informed Consent Forms for participating in the project. This study was approved by the Medical Ethics Committee of our hospital.

**Table 1:** General information of patients in the study and control groups

Item	Study group (*n* = 40)	Control group (*n* = 40)	*χ*^*2*^/Z	*P*
Gender				
Male	10(25.00)	9(22.50)	0.069	0.793
Female	30(75.00)	31(77.5)		
Age	16(15,17)	15.5(14,17)	−0.884	0.377
Education				
Elementary school	0(0.00)	2(5.00)	4.059	0.131
Middle school	26(65.00)	20(50.00)		
High school/ vocational school	14(35.00)	18(45.00)		
Course	7(5, 8)	6(5, 7)	−1.434	0.151
First episode				
Yes	28(70.00)	26(65.00)	0.228	0.633
No	12(30.00)	14(35.00)		
Only child				
Yes	32(80.00)	29(72.50)	0.621a	0.431
No	8(20.00)	11(27.50)		
Family relationship				
Harmonious	15(37.50)	12(30.00)	0.725	0.696
Divorced	15(37.50)	15(37.50)		
Parents separated	10(25.00)	13(32.50)		
Family history of despression				
Yes	2(5.00)	3(7.50)	0.215	0.643
No	38(95.00)	37(92.50)		

### Methods

Control Group: Mentalization-based therapy (MBT) was implemented for 8 weeks, once every 2 weeks (implementation phase week 2–7), for 2 h each session, scheduled every Sunday from 3:00 PM to 5:00 PM.

The intervention team included two chief psychiatrists and two specialized nurses each, and the intervention methods included family-oriented classroom guidance, experiential activities, role-playing, group discussions, games, and so on. The specific content is shown in Table 2.

**Table 2:** Specific Plan for MBFT

Time	Theme	Content
Week 1 (Starting phase)	Getting to know each other again	1. Briefly introduce the methods and precautions of the project to family members and patients; 2. Organize parents and children to sit in a circle and reacquaint themselves with each other on a parent–child basis; 3. Understand and analyze the parenting styles of each family and the problems in parent–child relationships 4. Establish training modules
Weeks 2–7 (implementation phase)	Conduct theoretical lectures and interactive training	1. Respect, understand, and acknowledge the child's feelings; 2. Teach parents and patients emotional management skills; 3. Encourage parents to praise patients to boost their self-confidence; 4. Guide family members and patients to learn communication skills; 5. Remind parents to listen to the patient's heart
Week 8 (Closing phase)	Conduct family education seminars	1. Encouraging parents to take the initiative to share their gains and questions; 2. Lead parents to summarize and reflect on past mistakes in parenting and communication; 3. Exchange contact information between parents and nursing staff for follow-up during the home period

Each module in the implementation phase is conducted in the form of classroom theoretical lectures, and interesting games, role-playing, and case studies are added according to the characteristics of each module, to help patients and family members master correct parenting and communication skills, and establish satisfactory parent–child relationships.

Study Group: the study group received mindfulness therapy based on group mindfulness and cognitive skills training for 8 weeks, in addition to the MBFT provided to the control group.

Only the patients who were in the recovery phase were included in this study; however, there was still a possibility of relapse in these patients. Adolescents with depression and suicidal ideation have a stronger sense of despair, poor family intimacy, and more extreme family situations compared to those without suicidal behavior [32]. Therefore, to create a comfortable and safe mindfulness environment for the subjects, a family member who could accompany them for an extended period was included as an intervention target during the intervention. To facilitate the formal implementation of the intervention, the subjects and family members were considered as a whole, and the 40 wholes were further divided into five groups. Each group was admitted to complete the in-hospital part of the intervention on a fixed day of the week from 3:00 PM to 5:00 PM (fixed dates such as: Group 1 on Monday, Group 2 on Tuesday, and so on), and the remaining 6 days were spent completing homework at home, supervised by the designated family member. After completing daily homework, family members also supervised the subject while they completed the daily homework record sheet in a standardized manner. Before implementing any new intervention in the clinic, a review of the homework for the past 6 days was conducted (except for the first week), and the subjects were guided to complete questionnaires to analyze whether there had been a change in their suicidal ideation in the short term and whether the intervention plan needed to be adjusted. No other interventions were employed in the participants during the study. The final efficacy analysis was based on the results of the questionnaire that was filled out by the participants after completing the 8-week homework. Initial questionairs were performed before the intervetions. These results were taken as the results for final questionaires. After each clinic intervention session, a summary of the current intervention content was conducted, and homework for the following six weeks was assigned (except for the first and eighth weeks). Specific mindfulness interventions were implemented by the psychiatric specialist nurses with national secondary-level psychological counseling qualifications. Before formally administering mindfulness interventions to the subjects, these specialist nurses attended a systematic mindfulness intervention course, engaged in daily self-practice, provided guidance for trainees’ individual mindfulness exercises during their probation and internship, and were supervised by personnel with higher qualifications. The specific intervention plan is shown in Table 3.

**Table 3:** Specific plan for mindfulness therapy based on group mindfulness and cognitive skills training

Time	Theme	In-hospital intervention content (with family members present)	Home-based homework (remaining 6 days, with the same family member)
Week 1	Identify auto-guidance	1. Patients and family members were paired to reacquaint themselves with each other, and nursing staff emphasized the confidentiality and precautions of this study to the patients and family members 2. Patients and family members both completed the "eating raisins" exercise and body scan (40–45 min)	1. Patients and family members both practiced body scan (40–45 min) every day 2. Patients and family members both completed one informal mindfulness exercise in daily life (such as mindful listening, mindful eating, and mindful cleaning) every day
Week 2	Help to deal with obstacles	1. Guide patients and family members to continue body scan exercise (40–45 min) 2. Guide patients and family members through the thought and feeling exercise and mindful breathing exercise (15 min)	1. Patients and family members both continued to practice body scan (40–45 min) and informal mindfulness exercises, adding mindful breathing exercise (15 min) every day; 2. Patients and family members shared enjoyable events from the past week that were triggered by each other and exchanged thoughts and feelings about these two events
Week 3	Mindfulness in motion	1. Lead patients and family members through mindfulness looking or listening, mindful breathing, and body awareness (15 min) exercises 2. Lead patients and family members through mindful walking (15 min) exercises with 3-min breathing spaces	1. Patients and family members both practiced mindfulness breathing and body awareness exercises (15 min) on odd-numbered days, and practiced body scan exercise (40–45 min) on even-numbered days 2. Patients and family members both practiced regular 3-min breathing spaces three times a day 3. Patients and family members described an unpleasant event triggered by each other in the past week and exchanged their views and suggestions about these two events
Week 4	Living in the present	1. Lead both patients and family members to practice mindfulness breathing and body awareness (15 min) and mindfulness walking (15 min) 2. Conduct a group education session within the group, discuss individual depressive symptoms, help patients and family members correctly identify negative thoughts, and allow family members to share past parenting and communication methods for nursing staff to correct wrong methods and demonstrate correct ones 3. Practice 3-min breathing space	1. Patients and family members both practiced mindfulness breathing and body awareness (15 min) and mindfulness walking (15 min) every day 2. Patients and family members both continued to practice regular 3-min breathing space 3. Patients and family members both tried to independently practice responsive 3-min breathing space (e.g., under stress or when upset)
Week 5	Accept, Allow, and Go with the Flow	1. Lead patients and family members in mindfulness breathing and body awareness (15 min) exercises 2. Conduct a group discussion on "cultivating an attitude of “allowing nature to take its course.” 3. Practice responsive 3-min breathing space	1. Patients and family members both continued mindfulness breathing and body awareness exercises (15 min), with audio assistance on odd-numbered days and in a quiet natural state on even-numbered days 2. Patients and family members both continued to practice regular 3-min breathing space and tried to complete them as many times as possible
Week 6	Thoughts are not facts	1. Lead patients and family members through mindfulness breathing and body awareness exercises (15 min) 2. Explore the patient's thoughts and feelings 3. Practice regular 3-min breathing space	Same as Week 5
Week 7	How to best take care of yourself	1. Lead patients and family members in mindfulness breathing and body awareness exercises (15 min) 2. Guide patients and family members to correctly identify signs of depression relapse, and jointly formulate response plans 3. Regular 3-min breathing space exercise	1. Patients and family members both chose one of the formal mindfulness exercise techniques they have learned to practice it for a long term 2. Patients and family members continued the practice as in Step 2 of Week 5 3. Patients and family members worked together to improve and refine the early warning plan for depression
Week 8	Applying learned techniques to future moods	1. Lead patients and family members through body scan exercise (40–45 min) 2. Review and summarize past courses 3. Develop a long-term, home-based mindfulness practice plan 4. Exchange contact information between family members and nursing staff for follow-up and allow family members to seek help at any time 5. Remind family members to bring patients to the hospital to complete psychological status and suicidal ideation tests after completing 6 days of homework this week	1. A joint practice plan was developed for patients and their family members, incorporating mindfulness exercises that best suit them 2. Practice informal mindfulness exercises as much as possible 3. Randomly switch between the regular and responsive 3-min breathing space exercise based on both parties’ situations

The specific formal mindfulness exercises were as follows:

(1) Body scan meditation: The subjects lied down in a relaxed posture, closed their eyes, and following the instructions on the recording, sequentially scanned each part of their body; they carefully explored the sensations occurring in each part of their body at that moment, thereby establishing an intimate connection with their body. (2) Mindful breathing and body awareness: the subjects selected a sitting posture in which they felt most comfortable, keeping their backs straight without leaning on any object. They placed both feet on the floor, closed their eyes, and attentively perceived the sensations of their body against the ground. They focused on the lower abdomen during the natural rhythm of inhaling and exhaling, feeling the rise and fall of the abdomen. If, during this process, the subject felt their attention wandering away from the abdomen, they gently shifted and explored and then quickly redirected their attention to the ongoing breath. (3) Mindful walking: the subjects relaxed their heads and necks, opened their eyes, and walked at the slowest and most comfortable pace of their daily life. They paused at a random location and spent 1 min observing their standing posture, with their arms positioned in a manner comfortable to them. After 1 min, they lifted one foot’s heel forward while inhaling and placed that foot’s toes on the ground while exhaling. The other foot followed the same pattern of lifting and placing, and they continued this sequence until the exercise was completed. (4) 3-min breathing space: The subjects first reflected on their current state of body, thoughts, and emotions and then focused on the sensations generated in their body during each breath, paying close attention. Finally, during each inhale and exhale, they focused on the body part where any unusual sensations occurred. No interaction was observed in our study, which indicated the good compatibility with each other between these two therapy.

### Observation indicators

- (1) Psychological condition: Psychological health of both groups before and after the intervention was assessed using the Middle School Student Mental Health scale (MssMHs), developed by Professor Wang Jisheng from the Chinese Academy of Sciences [33]. The MssMHs comprises 10 subscales, namely obsessive–compulsive symptoms, paranoia, hostility, interpersonal tension and sensitivity, depression, anxiety, academic stress, maladaptation, emotional imbalance, and psychological imbalance, with each subscale having six items. Each item was scored using a 5-point Likert scale (1–5 points), with higher scores indicating poorer psychological conditions. The mean score of each subscale was used to assess the psychological symptoms: a mean score of < 2 indicated psychological health; a mean score ranging between ≥ 2 and < 3 indicated mild psychological problems; a mean score between ≥ 3 and < 4 indicated moderate psychological problems; a mean score between ≥ 4 and < 5 indicated severe psychological problems; and a mean score of 5 indicated very severe psychological problems. This scale had good reliability, with a Cronbach’s α value of 0.96 for the total scale [34, 35].
- (2) Suicidal ideation: Suicidal ideation before and after intervention in both groups was evaluated using the positive and negative suicide ideation (PANSI) scale developed by Liu [36, 37]. PANSI was developed to assess the frequency of suicidal ideation among adolescents and adults aged ≥ 14 years, which includes negative risks and protective factors. PANSI consists of two factors: six positive thoughts and eight negative thoughts. Examples of positive ideation projects include "feeling in control of most situations in life" and "feeling excited about doing well in school or work". Examples of negative ideation projects include "feeling very lonely or sad, wanting to commit suicide so you can end the pain" and "wanting to commit suicide because you feel like you have failed in life". PANSI assesses the protective and risk factors associated with suicidal ideation, including two dimensions (14 items in total): positive ideation (PANSI-PI, 6 items) and negative suicidal ideation (PANSI-NSI, 8 items). PANSI-NSI and PANSI-PI examined the frequency of specific negative thoughts (such as failure to accomplish important tasks) or positive thoughts (such as excitement about performing well in school or work) related to suicidal behavior. Participants used the Likert scale, ranging from 1 (meaning "no time") to 5 (meaning "most of the time"), to assess their frequency of experiencing suicidal ideation. The higher the score, the more positive or negative suicidal ideation, depending on the specific subscale of the project. Each item was rated on a 5-point Likert scale (1–5 points), with scores positively correlated with the frequency of suicide ideation. The items under negative suicidal ideation were positively scored, while those under positive suicidal ideation were negatively scored. Total suicidal ideation score was the sum of total negative and positive suicidal ideation dimension scores, with higher scores indicating a higher degree of suicidal ideation. The scale had good reliability, with a Cronbach’s α coefficient of 0.91 for the total scale [38].

### Statistical analysis

After the data were checked, verified, and saved in a database, they were statistically analyzed using SPSS 21.0 software. Subjects’ general information, MssMHs scores, and PANSI scores were first tested for normality using a Shapiro–Wilk test. Cronbach’s alpha coefficients, item-total subscale correlations, and repeatability of the scale were used to establish the internal consistency of the scale. Internal consistency of items was evaluated with Cronbach's alpha coefficients. Correlations were used to analyze the construct validity of PANSI. The discriminative validity, specificity and sensitivity of the scales data were analyzed using logistic regression analysis. Normally distributed metric data are represented as the mean ± standard deviation ($\overline{x} \, \pm \,s$) and were tested using an independent sample t-test. Non-normally distributed metric data are represented as *M*_*50*_ (*P*_*25*_, *P*_*75*_) and analyzed using the *Mann–Whitney U test*, which is a non-parametric test. Count data are expressed as [*n *(%)] and analyzed using the χ^2^ test. The Mann Whitney U test is used to compare quantitative data between two groups. Kruskal Wallis one-way ANOVA is used to compare two or more quantitative variables. The Dunn test is used as a post hoc test in the analysis of variance. The chi square test is used to compare demographic data between two groups. The significance level of the test was α = 0.05, with *P* < 0.05 indicating statistical significance.

## Results

### Comparison of MssMHs scores before and after intervention in both groups

MssMHs scores did not differ significantly between the two groups before intervention (*P* < 0.05). After intervention, the MssMHs scores of both groups were lower, and the study group scored less than the control group (*P* < 0.05). The difference in MssMHs scores before and after intervention in the study group was higher than that in the control group (*P* < 0.05). In terms of obsessive–compulsive symptoms, paranoia, interpersonal tension and sensitivity, emotional imbalance nad psychological imbalance, the effects were significantly different. Details are presented in (Table 4).

**Table 4:** Comparison of MssMHs scores before and after intervention in both groups [$\overline{x} \, \pm \,s$, *M50 (P*_*25*_, *P*_*75*_*)*, points]

Item	Time	Study group (*n* = 40)	Control Group (*n* = 40)	*Z/t*	*P*
Obsessive–compulsive symptoms	Before intervention	4(3,4)	4(3,4)	−0.108	0.914
	After intervention	2(1,2)	3(2,3)	−5.038	< 0.001
	Difference	2(2,2.75)	1(0,2)	−4.183	< 0.001
Paranoia	Before intervention	3(3,4)	3(3,4)	−1.671	0.095
	After intervention	1(0,1)	2(1.25,3)	−5.507	< 0.001
	Difference	3(2.3)	1.5(1,2)	−3.824	< 0.001
Hostility	Before intervention	3(3,4)	3(3,4)	−1.687	0.092
	After intervention	1(1,2)	2(2,3)	−4.465	< 0.001
	Difference	2(1,3)	1(1,2)	−2.587	0.010
Interpersonal tension and sensitivity	Before intervention	3(3,4)	3(3,4)	−0.578	0.563
	After intervention	2(2,2)	2(2,3)	−5.783	< 0.001
	Difference	2(1,2)	1(0,1)	−4.926	< 0.001
Depression	Before intervention	3.5(3,4)	3(3,4)	−0.694	0.488
	After intervention	1(1,2)	2(2,2.75)	−4.151	< 0.001
	Difference	2(1.25,3)	1(1,2)	−3.561	< 0.001
Anxiety	Before intervention	4(3,4)	4(3,4)	−0.725	0.469
	After intervention	2(1,2)	3(2,3)	−4.828	< 0.001
	Difference	2(1,3)	1(1,2)	−3.445	0.001
Academic stress	Before intervention	4(3,4)	4(3,4)	−0.598	0.550
	After intervention	2(1,2)	3(2,3)	−4.495	< 0.001
	Difference	2(1.25,2)	1(0,2)	−3.431	0.001
Maladaptation	Before intervention	3(3,4)	3(3,4)	−0.738	0.460
	After intervention	1(0,1)	2(1,2)	−4.76	< 0.001
	Difference	2.5(2,4)	2(1,2)	−3.071	0.002
Emotional imbalance	Before intervention	4(3,4)	3.5(3,4)	−1.209	0.227
	After intervention	2(1,2)	3(2,3)	−4.785	< 0.001
	Difference	2(1.25,3)	1(0,2)	−4.066	< 0.001
Psychological imbalance	Before intervention	3(3,4)	3(3,4)	−1.518	0.129
	After intervention	1(1,1)	2(1.25,3)	−5.401	< 0.001
	Difference	2.5(2,3)	1(0.25,2)	−4.810	< 0.001
Total score	Before intervention	36.20 ± 2.07	36.03 ± 2.04	−0.381	0.704
	After intervention	13.23 ± 2.43	22.85 ± 2.43	17.685	< 0.001
	Difference	22.98 ± 3.27	13.18 ± 3.43	−13.072	< 0.001

Normally distributed metric data are represented as the mean ± standard deviation ($\overline{x}\, \pm \,s$) and were tested using an independent sample t-test. Non-normally distributed metric data are represented as *M*_*50*_ (*P*_*25*_, *P*_*75*_) and analyzed using the *Mann–Whitney U test*, which is a non-parametric test

### Comparison of PANSI scores before and after intervention in both groups

There were no statistically significant differences in the PANSI scores between the two groups before intervention (*P* < 0.05). After intervention, both groups had lower PANSI scores, with the study group scoring lower than the control group (*P* < 0.05). The absolute difference in PANSI scores before and after intervention in the study group was higher than that in the control group (*P* < 0.05). The results are presented in detail in Table 5.

**Table 5:** Comparison of PANSI scores before and after intervention in both groups ($\overline{x} \, \pm \,s$, points)

Item	Time	Study group (*n* = 40)	Control group (*n* = 40)	*T*	*P*
Negative suicidal ideation	Before intervention	10.20 ± 2.42	10.08 ± 2.23	−0.TE240	0.811
	After intervention	7.98 ± 3.17	9.55 ± 2.83	2.346	0.021
	Difference	2.23 ± 3.86	0.53 ± 3.57	−2.044	0.044
Positive suicidal ideation	Before intervention	16.90 ± 2.46	17.23 ± 2.47	0.590	0.557
	After intervention	23.28 ± 1.92	19.83 ± 1.85	−8.176	< 0.001
	Difference	−6.38 ± 3.19	−2.55 ± 2.73	5.763	< 0.001
Total score	Before intervention	27.10 ± 3.27	27.30 ± 3.43	0.267	0.790
	After intervention	31.25 ± 3.15	29.38 ± 3.51	−2.512	0.014
	Difference	−4.15 ± 3.85	−2.03 ± 4.44	2.286	0.025

Normally distributed metric data are represented as the mean ± standard deviation ($\overline{x} \, \pm \,s$) and were tested using an independent sample t-test. Non-normally distributed metric data are represented as *M*_*50*_ (*P*_*25*_, *P*_*75*_) and analyzed using the *Mann–Whitney U test*, which is a non-parametric test

## Discussion

### Positive impact of mindfulness therapy combined with MBFT on the mental health of adolescents with depressive disorder

Although a prior literature reported that MBI may be helpful in treating anxiety and emotional disorders, Toneatto and Nguyen concluded that MBI does not have a reliable effect on these issues [39]. MBI was originally developed to treat adults diagnosed with borderline personality disorder (BPD), and it directly addresses the fragile psychological abilities that are core features of BPD [22] and other complex pathologies. In the adult BPD population, MBI reduces self harm, emotional distress, and hospital stay compared to normal treatment, and improves social function, with continuous improvement at 18 months [18]. In this study, the impact of mindfulness therapy combined with MBFT on the mental health of adolescents with depressive disorder were examined. Compared to the control group, the mental health of patients in the study group was significantly better after intervention. These findings are in agreement with those reported by Shao et al. [40] and others, who found that positive psycho-cognitive intervention can improve the mental health of middle school students. According to a study by Massachusetts General Hospital in 2010, this may be attributed to the amygdala, which, as the brain’s emotional center, is one of the brain structures that is most closely correlated to the perception of stress and anxiety and plays a crucial role in regulating stress responses. An 8-week mindfulness intervention not only promoted psychological changes in the patients but also triggered structural changes in their brain. As the perception of stress decreased, the gray matter density of the amygdala gradually decreased, and the gray matter density of the hippocampus increased [41]. Therefore, while designing the experimental approach for this study, emphasis was placed on guiding subjects to feel and accept themselves in the present. When strong emotions arose, the subjects were encouraged to feel them and accept them, instead of reacting and showing extreme behavior. The subjects were also encouraged to actively share their thoughts with the people around them. This process not only prevented strong emotional fluctuations but also helped adolescents develop expressive and communication skills, alleviate depressive emotions, and ultimately, effectively shift and release their attention from negative events in the present [42]. Over time, the mental health of the adolescents showed improvement. Moreover, medical staff and family members accompanied the subjects throughout the mindfulness intervention process, which enabled them and their family members to raise their concerns at any time and seek guidance by medical staff and learn the correct emotional processing and communication methods [43].

### Regulating effect of the combined mindfulness therapy and MBFT on suicidal ideation in adolescents with depressive disorder

MBFT is associated with several positive benefits that may have an impact on the risk of suicide. MBFT can reduce factors such as impulsivity and depressive symptoms, which are associated with a higher risk of suicide. In addition, it also improves various cognitive processes such as executive function and attention, and increases metacognition and self-awareness [44]. The hypothesis that MBFT training increases the formal practice of better cognitive processes and less symptomatic lifestyles, thereby reducing the risk of suicide seems very reasonable. Learning mindfulness skills is an important mediator in reducing depressive symptoms [45]. Herein, the combined mindfulness therapy and MBFT was applied to examine the suicidal ideation in adolescents with depressive disorder. In our study, we found that both groups had lower PANSI scores after intervention, with the study group scoring lower than the control group (*P* < 0.05). The results from this study showed that the study group exhibited significantly lower negative suicidal ideation scores but significantly higher positive suicidal ideation scores than the control group after intervention. Meanwhile, the results also show that the absolute difference in PANSI scores before and after intervention in the study group was higher than that in the control group (*P* < 0.05), indicating a more profound clinical improvement. These results indicated the effecacy of the proposed therapy in reducing suicidal ideation. This result is consistent with the findings of McCauley E and others [46], who has found that dialectical behavior therapy can reduce self harm and suicidal ideation in adolescents with high suicidal tendencies. This may also be attributed to the intentional inclusion of a family member in this study and requiring them to complete an 8-week mindfulness intervention with the subjects. This may have a positive impact on the relationship between the subjects and their family members, bringing them closer and providing them with an opportunity to get to know each other's inner world up close. It helps family members correct their past mistakes in parenting and communication methods, and this companionship provides subjects with sufficient family support, psychological support, and a sense of security. This in turn leads to the establishment of better parent–child relationships, which can weaken the suicidal ideation or behavior of adolescent depression patients. In addition, mindfulness walking and 3 min of breathing space can help patients and their families master concepts that they do not judge or accept, allowing them to have a deeper understanding of themselves and each other. They can discover their own and each other’s strengths, more easily achieve self-satisfaction and external recognition, and generate positive emotions [47]. Family members also supervised subjects to complete the intervention plans on time. Wang et al. [48] found that adolescents with suicidal thoughts undergo significant changes over time, and thus, regular assessments are necessary to make timely adjustments to the intervention plan according to the subjects’ current mental state. Compared to conventional psychological interventions, the proposed program also includes stage goals and mood measurements, which can ensure acceptance by subjects and effectively reduce their suicide ideation.

Some limitations and advantages of current study should be mentioned. Firstly, and most importantly, this study is only based on a small sample of patients. Because of this, this study may be more susceptible to false influences and the generalizability of its findings is less certain. A potential challenge brought by small numbers is that randomization is more likely to fail to produce groups that are comparable in all important aspects. Another limitation involves the lack of an active control group. Furthermore, we did not measure the loyalty of therapy in terms of compliance and capability. The research design does not allow for clear causal inference. Suicide ideation is measured by a single item. Although this is a common practice in the research field, the use of psychological measurement scales for suicidal ideation is advisable and may improve the reliability of evaluations in future surveys. The advantages include a large sample size and sufficient ability to analyze the interaction effects over time. Due to the fact that MBI was originally developed to prevent the recurrence of depression, the long-term stability of the results of this study is particularly interesting. Therefore, future research may investigate the impact of MBI on self-reported suicidal ideation over a longer period of time.

## Conclusion

This study implemented the combination of mindfulness therapy and MBFT for adolescents with depression. This approach improved the mental health of adolescents with depression, reduced their negative suicidal ideation, and helped them better cope with their current lives. However, this study has some limitations; the sample size was relatively small, and there was no long-term follow-up after the intervention. Therefore, it remains unclear whether the proposed method addresses the drawback of having only a short-term effect without long-term efficacy, as revealed in previous studies. In addition, the study only involved intervention and did not conduct in-depth research on the correlations between different parts of the intervention plan and the related indicators. Hence, the most effective and helpful aspect of mindfulness therapy for behavioral changes in patients, among body scan meditation, mindful breathing, body awareness, mindful walking, and 3-min breathing space, remains to be determined. In addition, this study primarily used subjective indicators to monitor changes in patient mental health and suicidal ideation, excluding objective indicators such as neurological function, cortisol, cortical or gray matter density. Therefore, future research should expand the sample size, continuously improve intervention plans, enrich observation indicators, and provide reference value for the comprehensive promotion of mindfulness intervention and MBFT in similar situations such as psychological disorders and mental illnesses. It is also worth noted that more training for clinician who are going to help with the implementation of the therapy are needed in the future before the combined therapy could be utilized in clinical settings.

## Data Availability

All data generated or analysed during this study are included in this published article.

## Abbreviations

MBFT: Mentalization-based family therapy
ICD: International Classification of Diseases
PM: Post meridiem
MssMHs: Middle School Student Mental Health scale
PANSI: Positive and negative suicide ideationz
